# Exploring spin-polarization in Bi-based high-*T*_*c*_ cuprates

**DOI:** 10.1038/s41598-023-40145-1

**Published:** 2023-08-18

**Authors:** Hideaki Iwasawa, Kazuki Sumida, Shigeyuki Ishida, Patrick Le Fèvre, François Bertran, Yoshiyuki Yoshida, Hiroshi Eisaki, Andrés F. Santander-Syro, Taichi Okuda

**Affiliations:** 1 Institute for Advanced Synchrotron Light Source, National Institutes for Quantum Science and Technology, Sendai, 980-8579 Japan; 2 Synchrotron Radiation Research Center, National Institutes for Quantum Science and Technology, Hyogo, 679-5148 Japan; 3 QST Advanced Study Laboratory, National Institutes for Quantum Science and Technology, Chiba, 263-8555 Japan; 4 Hiroshima Synchrotron Radiation Center, Hiroshima University, Hiroshima, 739-0046 Japan; 5 Materials Sciences Research Center, Japan Atomic Energy Agency, Hyogo, 679-5148 Japan; 6 Research Institute for Advanced Electronics and Photonics, National Institute of Advanced Industrial Science and Technology, Ibaraki, 305-8568 Japan; 7 SOLEIL Synchrotron, L’Orme des Merisiers, Départementale 128, 91190 Saint-Aubin, France; 8 Institut des Sciences Moléculaires d’Orsay, CNRS, Université Paris-Saclay, 91405 Orsay, France

**Keywords:** Superconducting properties and materials, Electronic properties and materials

## Abstract

The role of spin–orbit interaction has been recently reconsidered in high-$T_{c}$ cuprates, stimulated by the recent experimental observations of spin-polarized electronic states. However, due to the complexity of the spin texture reported, the origin of the spin polarization in high-$T_{c}$ cuprates remains unclear. Here, we present the spin- and angle-resolved photoemission spectroscopy (ARPES) data on the facing momentum points that are symmetric with respect to the $\Gamma$ point, to ensure the intrinsic spin nature related to the initial state. We consistently found the very weak spin polarization only along the nodal direction, with no indication of spin-splitting of the band. Our findings thus call for a revision of the simple application of the spin–orbit interaction, which has been treated within the standard framework of the Rashba interaction in high-$T_{c}$ cuprates.

## Introduction

High-temperature (High-$T_c$) cuprate superconductors are generally considered to be systems where electron correlations are the dominant interaction. While prior studies have reported anomalous spin behavior in these materials^1,2^, the spin–orbit interaction has been largely overlooked or treated as a minor perturbation, leading to insignificant effects on the electronic ground state of these materials^3,4^. Conversely, recent spin- and angle-resolved photoemission spectroscopy (ARPES) measurements on high-$T_c$ cuprates have shown significant spin polarization, implying the presence of spin–momentum locking induced by the spin–orbit interaction^5^. This has prompted theoretical investigations into the role and consequences of the Rashba-type spin–orbit interaction in cuprate superconductors, highlighting the potential importance of this interaction in high-$T_c$ cuprates^6–8^. However, the driving force behind the reported spin-polarized electronic states remains unclear, perhaps due to their unique spin texture, which involves a sign reversal of spin polarization from the node to off-nodes, even within one Fermi surface. This texture cannot be explained by a simple Rashba-like helical spin texture, leading to an empirical and peculiar explanation, which requires two types of helical spin textures with opposite rotational directions, centered at the Brillouin zone center and corners^5^. Despite the complexity of this behavior, the available experimental information is limited, as the reported reversal spin texture was only examined within the same one-quarter Fermi surface.

In this work, we present spin-resolved ARPES data obtained from Bi-based high-$T_c$ cuprates. The data were collected from symmetric momentum points with respect to the $\Gamma$ point, in order to capture the intrinsic spin nature of the initial state. Our results consistently revealed very weak spin polarization only along the nodal (diagonal) direction in the Brillouin zone. We found spin polarizations from a single band, with no indication of the spin-splitting of the band. Our results thus suggest the another mechanism should be involved with the observed spin-polarization beyond the standard Rashba spin–orbit interaction.

## Experimental details

**Figure 1:**
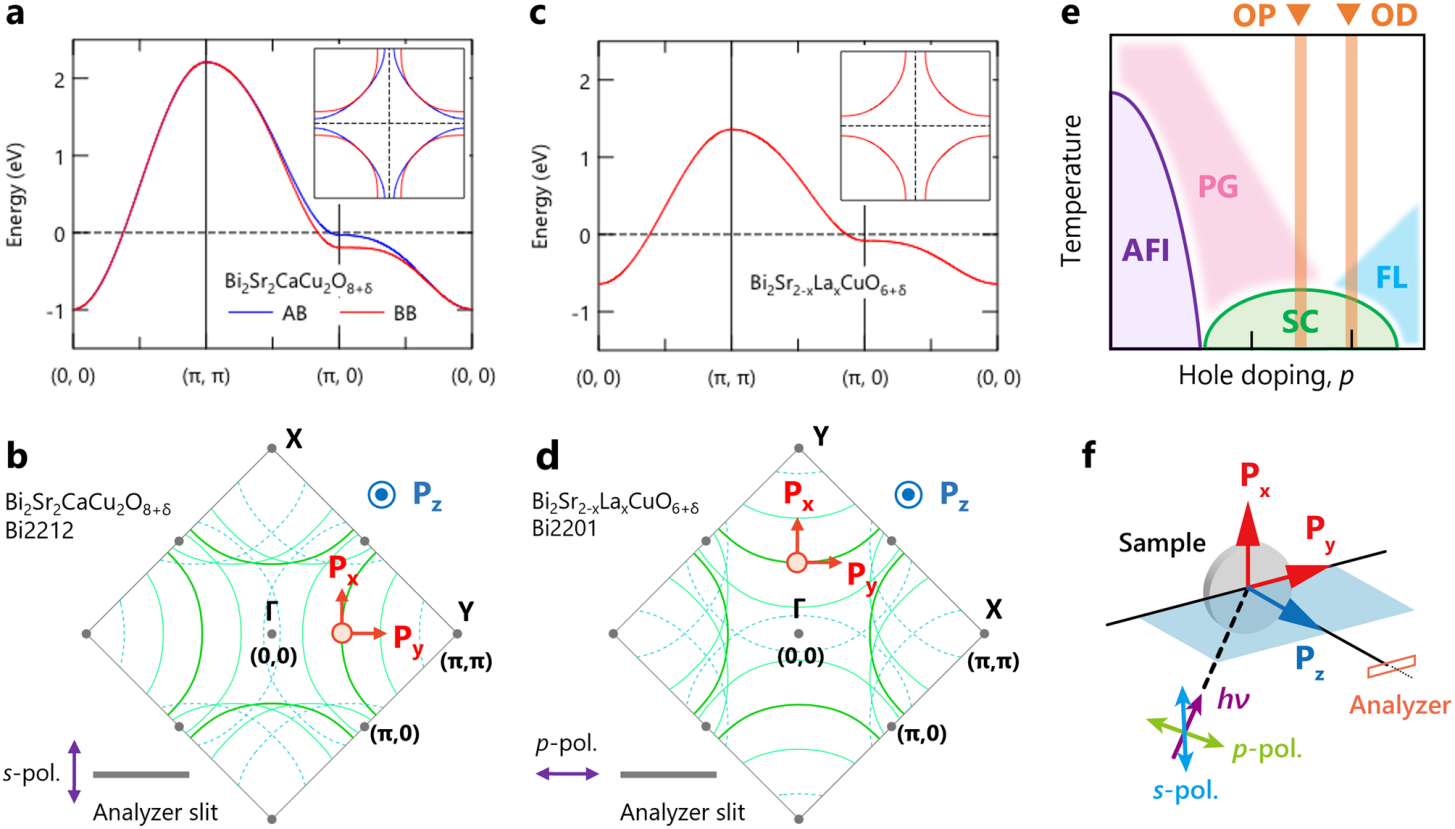
Overview of the electronic structure of Bi-based high-$T_{c}$ cuprates and the experimental configuration of spin-resolved ARPES measurements. (**a**,**b**) and (**c**,**d**) Electronic structure of Bi_2_Sr_2_CaCu_2_O$_{8+\delta }$ (Bi2212) and Bi_2_Sr$_{2-x}$La$_x$CuO$_{6+\delta }$ (Bi2201), respectively, together with the Fermi surface (inset). They were calculated using the tight-binding (TB) model, as described elsewhere^11,12^. (**e**) Schematic phase diagram, where doping levels measured in this study, optimal-dope and over-dope (OP and OD), are highlighted. The AFI, PG, SC, and FL denote the antiferromagnetic insulating states, pseudogap states, superconducting states, and Fermi liquid states, respectively. (**f**) Experimental configuration of the present spin-resolved ARPES measurements. The polarization direction of incident light is perpendicular (*s*-polarization) or parallel (*p*-polarization) to the analyzer slit. The in-plane spin components ($P_x$ and $P_y$) lay in the *ab*-plane of the sample surface, while the out-of-plane spin component ($P_z$) is along the *c*-axis.

High-quality single crystals of optimally doped (OP) and overdoped (OD) Bi_2_Sr_2_CaCu_2_O$_{8+\delta }$ (Bi2212) and OD Bi_2_Sr$_{2-x}$La$_x$CuO$_{6+\delta }$ (Bi2201) were grown by the traveling-solvent floating-zone technique^9^. The superconducting transition temperature ($T_{c}$) of these samples is 90 K and 75 K for OP- and OD-Bi2212, and 27 K for OD-Bi2201. Note that the OP-Bi2212 is slightly overdoped but labeled as OP for simplicity. ARPES and spin-resolved ARPES experiments were performed at CASSIOPEE beamline at SOLEIL synchrotron by a hemispherical electron analyzer (MBS A1) with spin detection using a FERRUM VLEED detector^10^. Present data were measured at 20 K using a photon energy of 30 eV and 50 eV with linear polarizations (vertical or horizontal) after cleaving the samples in situ in ultrahigh vacuum better than 9$\times$10$^{-11}$ mbar at 20 K. The experimental geometry and spin components in the spin-resolved ARPES experiments are depicted in Fig. 1f. The energy and angular resolution were set to be 40 meV and 1.2$^\circ$, respectively. It should also be noted that we calibrated the image intensity of the ARPES images and Fermi surface maps to improve visibility (see Supplementary Fig. 1), but we did not apply these calibrations to the spin-ARPES data. Therefore, our results and conclusions were not affected by these calibrations.

## Results

We have investigated spin-dependent electronic structures of Bi-based bilayer and single-layer cuprates: OD- and OP-Bi2212 with the superconducting transition temperature $T_{c}$=75 K and 90 K, respectively, and OD-Bi2201 with $T_c$=27 K. The doping levels of these cuprates are highlighted in a schematic phase diagram of hole-doped cuprate superconductors (Fig. 1e). Spin-resolved ARPES data were collected from Bi2212 and Bi2201 along (or parallel to) the $\Gamma -Y$ direction (see their electronic structures and Fermi surfaces, calculated by the tight-binding model^11,12^, as shown in Fig. 1a–d). Although the measurement direction is the same between Bi2212 and Bi2201, the experimental orientation is different by 90$^\circ$. Accordingly, the in-plane component of spin polarization $P_{x}$ ($P_{y}$), tangential to the Fermi surface, was examined with *s*-polarized light for Bi2212 while with *p*-polarized light for Bi2201, in order to keep essentially the same experimental condition. The spin polarization (*P*) is determined as the asymmetry between spin-up ($I_{\uparrow }$) and spin-down states ($I_{\downarrow }$), which is proportional to the difference in the scattered electron intensity between positively and negatively magnetized targets in the VLEED detector. To minimize effects due to instrumental asymmetries, four measurements were taken for each polarization direction under varied conditions, which included the reversal of both the magnetization direction and the electron spin direction. Then, the polarization was determined by $P=S^{-1}(I_{+}-I_{-})/(I_{+}+I_{-})$, where both $I_{+}$ and $I_{-}$ were obtained by calculating a geometric mean of the four measurements, and *S* is the Sherman function, which we assumed to be 0.2. As shown in Fig. 1a, the splitting between bonding and antibonding bands (AB and BB) is not visible near the nodal direction (${\Gamma }Y/{\Gamma }X$) in Bi2212. In the present study using conventional photon energies (30 eV and 50 eV), the two bands were not resolved and were observed mixedly like a single band. It is important to note that the dominant band character changes; it is expected to be AB for 30 eV and BB for 50 eV due to matrix element effects^13^. The experimentally observed electronic states of Bi2212 and Bi2201 consist not only of the single main band (MB) but also of the diffraction replicas (DRs) or umklapp bands, due to photoelectron diffraction^14,15^, as seen in Fig. 1b,d. Since the measured momentum locations are along or near the nodal region, where the MBs and DRs are well separated, the spin polarization of each band can be examined without interference due to the presence of multiple bands.

**Figure 2:**
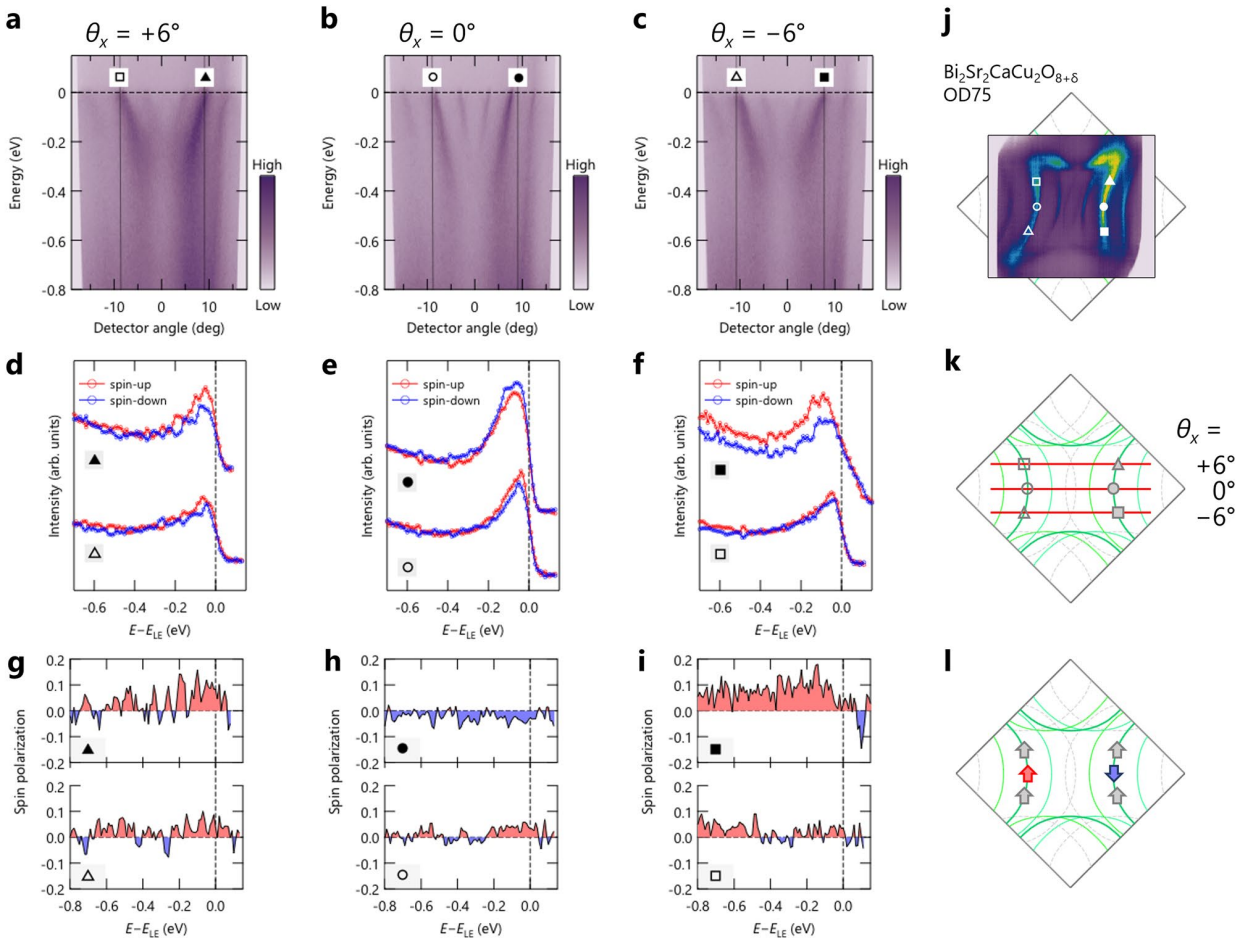
Spin-resolved ARPES results from overdoped Bi2212 taken at 30 eV. (**a**–**c**) Nodal and off-nodal ARPES images taken along the cuts at different deflector angles ($\theta _{x}=0^{\circ }$ and $\theta _{x}=\pm\,$6$^{\circ }$), as indicated in (**j**) the schematic Fermi surface. (**d**–**f**) and (**g**–**i**) Spin-resolved EDCs and spin polarization, respectively, at Fermi momenta indicated by markers in (**a**–**c**), where their energies were rescaled with respect to the energy of the spectral leading edge ($E_{\text {LE}}$). (**j**–**l**) Experimental Fermi surface, measurement locations, and spin textures are respectively overlaid on the calculated Fermi surface by the tight-binding model.

Figure 2 summarizes the main results of this work, displaying the spin polarization and texture in overdoped Bi2212. The in-plane spin components $P_{x}$ were examined by the *s*-polarized 30 eV photons at three different momentum cuts, as shown in the spin-integrated ARPES data taken along the nodal direction (Fig. 2b) where the SC gap closes, and the off-nodal directions (Fig. 2a,c) where the SC gap opens by $\sim$10 meV. To simplify matters, all the figures presented in this work were calibrated with respect to the energy of the spectral leading edge ($E_{\texttt {LE}}$). Figure 2d–f shows the spin-resolved energy distribution curves (EDCs), where the red and blue curves indicate the spin-up and spin-down signals, respectively. It should be noted that we compare two pairs of spin-up and spin-down EDCs at symmetric Fermi momenta ($k_{\texttt {F}}$) with respect to the $\Gamma$ point, as indicated by the markers in Fig. 2j,k, where each filled and opened one makes a pair.

At first sight, a discernible intensity difference between spin-up and spin-down EDCs is evident for all the pairs, corresponding to 5–10$\%$ spin polarization (Fig. 2g–i). Notably, the sign of spin-polarization is flipped for the nodal direction (Fig. 2e), suggesting the intrinsic nature of the observed spin-polarization originated in the initial state. In contrast, the spin-polarization at off-nodal regions was not reversed with respect to the mirror plane, implying the extrinsic nature of the spin-polarization, such as the final state effect^16,17^. Figure 2l displays a spin texture based on the observed spin-polarization in this work, where the spin-polarization persists only along the nodal line.

**Figure 3:**
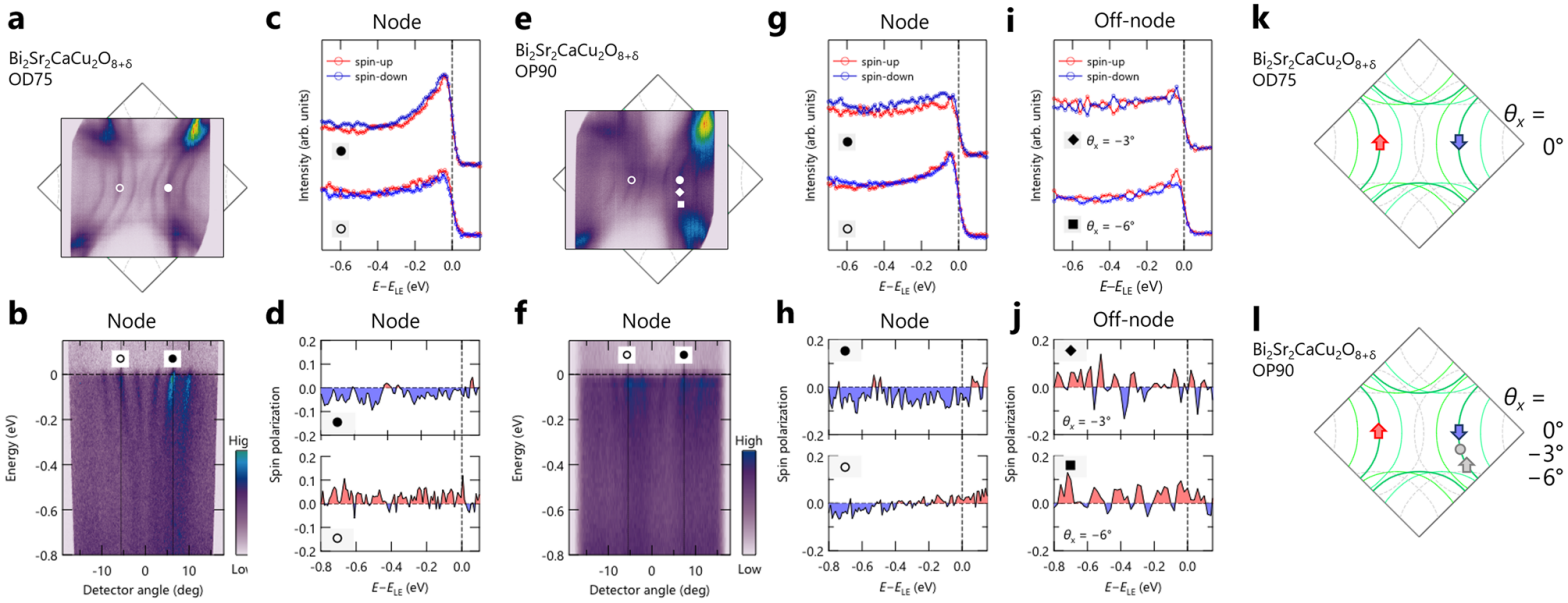
Spin-resolved ARPES results from overdoped and optimally-doped Bi2212 taken at 50 eV. (**a**) and (**b**) Experimental Fermi surface and nodal ARPES image of OD-Bi2212, respectively. (**c**) and (**d**) Spin-resolved EDCs and spin polarization of OD-Bi2212, respectively, at Fermi momenta indicated by markers in (**a**) and (**b**). (**e**) and (**f**) Same as (**a**) and (**b**), except that they are for OP-Bi2212. (**g**–**j**) Spin-resolved EDCs and spin polarization of OP-Bi2212 in the nodal and off-nodal regions, where their energies were rescaled with respect to the energy of the spectral leading edge ($E_{\text {LE}}$). Measured momentum locations are indicated by markers in (**e**). (**k**) and (**l**) Spin textures for OD- and OP-Bi2212, respectively.

To further explore the nature of the observed nodal spin polarization, we examined photon energy and doping dependence on the spin polarization. Figure 3 shows the spin-resolved ARPES results from the OD-Bi2212 and OP-Bi2212 using a different photon energy of 50 eV with *s*-polarization. Among the clear MBs and 1st DRs with the faint 2nd DRs, we focused on the MBs along the nodal line, as indicated in Fig. 3a,b. A small but clear reversal of the spin polarization across the $\Gamma$ point was observed, as shown in Fig. 3c,d. The spin polarization $P_x$ is about 5$\%$ near the Fermi energy, consistent with the results obtained by 30 eV (Fig. 2e,h). It should be noted that the observed band character changes with the photon energy, considering the matrix element effects^13^: the photoemission signals are expected to be dominated by the AB for 30 eV while the BB for 50 eV. Our results thus indicate the same spin texture for the BB and AB in Bi2212. The doping dependence of the spin polarization on MBs along the nodal and off-nodal region in OP-Bi2212 is shown in Fig. 3g–j. The measured momentum locations are indicated by markers in Fig. 3e,f. Along the nodal direction, we found the reversal spin polarization across the $\Gamma$ point, although the magnitude of spin polarization is asymmetric: $P_x$ is about 2$\%$ and 9$\%$ for positive and negative spin polarization, respectively. On the other hand, the spin polarization of the MB on the right-hand side changes its magnitude and sign as − 9$\%$, 0$\%$, and 3$\%$ from the nodal ($\theta _{x}=0^{\circ }$) to off-nodal ($\theta _{x}=-3^{\circ }$ and $\theta _{x}=-6^{\circ }$) region. This momentum-dependent sign reversal behavior is consistent with previous spin-resolved ARPES data^5^. However, comparing the spin textures that we obtained from OD-Bi2212 (Figs. 2l and 3k) and OP-Bi2212 (Fig. 3l), the presence of spin-polarization is validated only along the nodal direction from our data.

**Figure 4:**
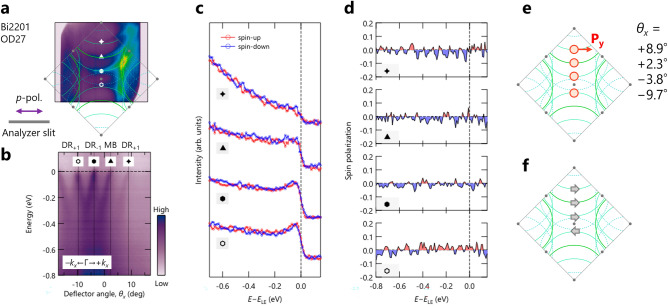
Spin-resolved ARPES measurements on overdoped Bi2201 taken at 30 eV. (**a**) and (**b**) Experimental Fermi surface and nodal ARPES image of OD-Bi2201, respectively. Note that the ARPES image was extracted vertically from the Fermi surface mapping data, and as a result, its horizontal axis is a function of the deflector angle. (**c**) and (**d**) Spin-resolved EDCs and spin-polarization of OD-Bi2201, respectively, at Fermi momenta indicated by markers in (**a**) and (**b**), where their energies were rescaled with respect to the energy of the spectral leading edge ($E_{\text {LE}}$). (**e**) and (**f**) Measured momentum locations and spin textures.

To place constraints on what is responsible for the observed nodal spin polarization, it is important to evaluate contributions from the local inversion symmetry breaking related to the two-CuO_2_ bilayer structure in Bi2212, as proposed in Ref.^5^. We thus examined the spin polarization on the single-layer OD-Bi2201, which should have different local electric fields around the CuO_2_ plane compared with those in Bi2212. Figure 4 shows the spin-resolved ARPES results from OD-Bi2201 taken at 30 eV. It should be noted that the sample orientation is different by 90$^\circ$ from those used for Bi2212, as shown in Figs. 2 and 3. Therefore, the target spin component is changed to $P_y$ and measured by the *p*-polarized light to keep the essentially same experimental conditions between the two systems. Similar to the observations in OD- and OP-Bi2212, the MB and 1st DRs are observed in OD-Bi2201, as shown in Fig. 4a,b. Here, the DR$_{\pm 1}$ (${\pm k_x}$) means that the DR band originated in the superstructure of the MB at the $\pm k_x$ side with the ± 1st-order superstructure with the vector *q* vector of $\pm\, 0.24$ (Ref.^15^). Thus, the DR$_{-1}$ (${+k_x}$) and DR$_{+1}$ (${-k_x}$) bands are only a pair across the $\Gamma$ point in this dataset. Figure 4c,d shows the observed spin-resolved EDCs and spin polarizations on these bands (see markers in Fig. 4a,b for measured $k_x$ locations). As anticipated from the small difference between the spin-up and spin-down EDCs, we did not observe any significant spin polarizations, as low as ± 2$\%$, in all the bands of Bi2201. On the other hand, one might notice a reversal spin-polarization for the DR$_{-1}$ (${+k_x}$) and DR$_{+1}$ (${-k_x}$) bands across the $\Gamma$ point, as seen in Fig. 4f, which might be suggestive of the intrinsic nature of spin states in Bi2201. However, further investigation is needed to confirm this observation, given the experimental accuracy and statistics limitations.

## Discussion

Recent spin-resolved ARPES study on the overdoped Bi2212 ($T_{c}$=58 K) by Gotlieb et al. reported several unique spin states^5^, which share both similarities and differences when compared with the present results.

Firstly, they observed significant spin polarization at the node, with a value of 20$\%$ near $E_{\texttt {F}}$ that increases to 40$\%$ at higher binding energy. In contrast, we consistently observed much smaller spin polarization, with a magnitude of about 5–10$\%$ at most. A possible and simple explanation for the observed difference could be attributed to variations in experimental conditions, such as doping levels and photon energy. The difference in doping might cause inconsistencies in spin polarization, but this is considered unlikely based on their follow-up report^18^, where spin polarizations of over 20$\%$ were observed for both under-doped and optimally-doped Bi2212 (with $p=0.12$ and $p=0.16$). From a technical viewpoint, a low excitation energy (6 eV) they used may provide higher bulk sensitivity^19,20^ but also introduce serious final-state effects^21^, compared to the conventional energy that we used. Another candidate for explaining a spin polarization difference may originate from phase shift effects during the photoemission process, which have been suggested as a possible explanation for the varying degrees of spin polarization, as 20$\%$ in Cu(111) and 10$\%$ in Bi2212 and Bi2201^22,23^. Note that the phase shift effects depend on the experimental conditions, such as the experimental geometry, photon energy, light polarization, and band symmetry because they are caused by the interference between the photoemission matrix elements for two transitions with different phases from an initial state to a final state^22^. Given the above considerations, almost identical conditions are necessary for both the material and experimental sides, to compare the magnitude of the spin polarization quantitatively.

Secondly, the spin texture was proposed to be a combination of two types of Rashba-like helical textures, centered at the Brillouin zone center ($\Gamma$ point) and zone corners (*Y*/*X* points). This complex spin texture was necessary to reproduce the peculiar momentum-dependent behavior, where the degree and sign of the spin polarization changed along the Fermi surface from the node to off-nodal points. In our present data (Fig. 2), such a reversal of spin polarization was observed on the one-quarter Fermi surface, though it was not consistently detected on another Fermi surface that is symmetric with respect to the $\Gamma$ point. Including present and existing spin-resolved ARPES studies, it is difficult to capture the spin texture of Bi-based cuprates by relying on information from limited *k* points. To address this issue, it is necessary to perform more comprehensive spin-resolved ARPES measurements. In particular, the in-plane two-dimensional spin components, parallel and perpendicular to the Fermi surface, should be examined along the pair of whole Fermi surfaces with respect to the $\Gamma$ point.

It should also be noted that a weak spin-polarization was recently reported by spin-resolved ARPES measurements on nearly optimally-doped Bi2212 ($T_{c}$ = 90 K), using a Xe lamp (8.43 eV)^24^. They argued the electronic spin symmetry of Bi2212 becomes $C_2$ by breaking $C_4$ rotational symmetry based on the anisotropy by 4$\%$ in the magnitude of spin polarization between the nodes along the ${\Gamma }Y$ and ${\Gamma }X$ lines, which should be equivalent with respect to the $C_4$ symmetry. However, our present data showed the anisotropy by 7$\%$ between $\pm k_{\texttt {F}}$ even along the same ${\Gamma }Y$ line (Fig. 2h). Therefore, discussion regarding the absolute value of spin polarization can only be established with careful attention to detail and the use of sufficiently accurate data. Considering the very weak spin polarization observed in our experiment, future work will require higher signal-to-noise ratios and sufficient statistical accuracy to quantitatively evaluate spin polarization in the Bi-cuprate systems.

Lastly, we will provide comments on the origin of spin polarization. It was initially proposed that local inversion-symmetry breaking in Bi2212 could lead to the emergence of a non-zero electric field between the bilayer CuO_2_ planes^5^. This, in turn, could induce a Rashba-like helical spin texture with two opposite rotation directions for the upper and lower CuO_2_ planes. However, this scenario alone cannot fully explain the presence of spin polarization in single-layer Bi2201, which has been reported previously^5,18,23^. Alternatively, the local symmetry breaking due to short-range distortions of the CuO octahedra is likely a possible common source of spin polarization in cuprate systems, as proposed in Ref.^18^. However, regardless of the possible sources that may give rise to a local electric field, a fundamental problem still remains as long as the effects are treated by Rashba-type spin–orbit interactions. So far, present and existing spin-resolved ARPES results have found no signature of a band splitting other than the bilayer splitting. It is thus apparent that further theoretical work is needed to go beyond the conventional framework of Rashba-type spin–orbit interactions^6–8^, which cannot explain the current results where no Rashba-like band-splitting is observed.

In summary, our spin-resolved ARPES measurements on OD- and OP-Bi2212 and OD-Bi2201 have revealed very weak spin-polarization only along the nodal direction consistently, as evidenced by examining the spin-polarization at both positive and negative wave vectors in the pair of one-quarter Fermi surfaces facing the $\Gamma$ point. Given the complex electronic and spin states observed in Bi-based cuprates, it is crucial for future studies to explore spin-polarization across a wider momentum space, encompassing the full Fermi surfaces. Such an endeavor would provide new insights into the role of spin–orbit interaction as well as the interplay between spin–orbit interaction and unconventional superconductivity in high-$T_c$ cuprates.

### Supplementary Information

Supplementary Figure S1.

## Data Availability

The datasets used and/or analysed during the current study available from the corresponding author on reasonable request.
