# Supplementary material for: Exploring spin-polarization in Bi-based high-Tc cuprates
Source: Sci Rep. 2023 Aug 18;13:13451. doi: 10.1038/s41598-023-40145-1 (PMC10439128; doi:10.1038/s41598-023-40145-1)
Supplement: Supplementary file 1 — Supplementary Figure S1. [file 41598_2023_40145_MOESM1_ESM.pdf]

Supplementary Information:
Exploring Spin-Polarization in Bi-Based High-T_c Cuprates

Hideaki Iwasawa[1,2,3,4,*], Kazuki Sumida[5], Shigeyuki Ishida[6], Patrick Le Fèvre[7], François Bertran[7], Yoshiyuki Yoshida[6], Hiroshi Eisaki[6], A. F. Santander-Syro[8], Taichi Okuda[4]

[1]*Institute for Advanced Synchrotron Light Source, National Institutes for Quantum Science and Technology, Sendai 980-8579, Japan*
[2]*Synchrotron Radiation Research Center, National Institutes for Quantum Science and Technology, Hyogo 679-5148, Japan*
[3]*QST Advanced Study Laboratory, National Institutes for Quantum Science and Technology, Chiba 263-8555, Japan*
[4]*Hiroshima Synchrotron Radiation Center, Hiroshima University, Hiroshima 739-0046, Japan*
[5]*Materials Sciences Research Center, Japan Atomic Energy Agency, Hyogo 679-5148, Japan*
[6]*Electronics and Photonics Research Institute, National Institute of Advanced Industrial Science and Technology, Ibaraki 305-8568, Japan*
[7]*SOLEIL Synchrotron, L'Orme des Merisiers, Départementale 128, F-91190 Saint-Aubin, France*
[8]*Université Paris-Saclay, CNRS, Institut des Sciences Moléculaires d'Orsay, 91405, Orsay, France*

*iwasawa.hideaki@qst.go.jp

Contents

Supplementary Figure S1

Supplementary Figure

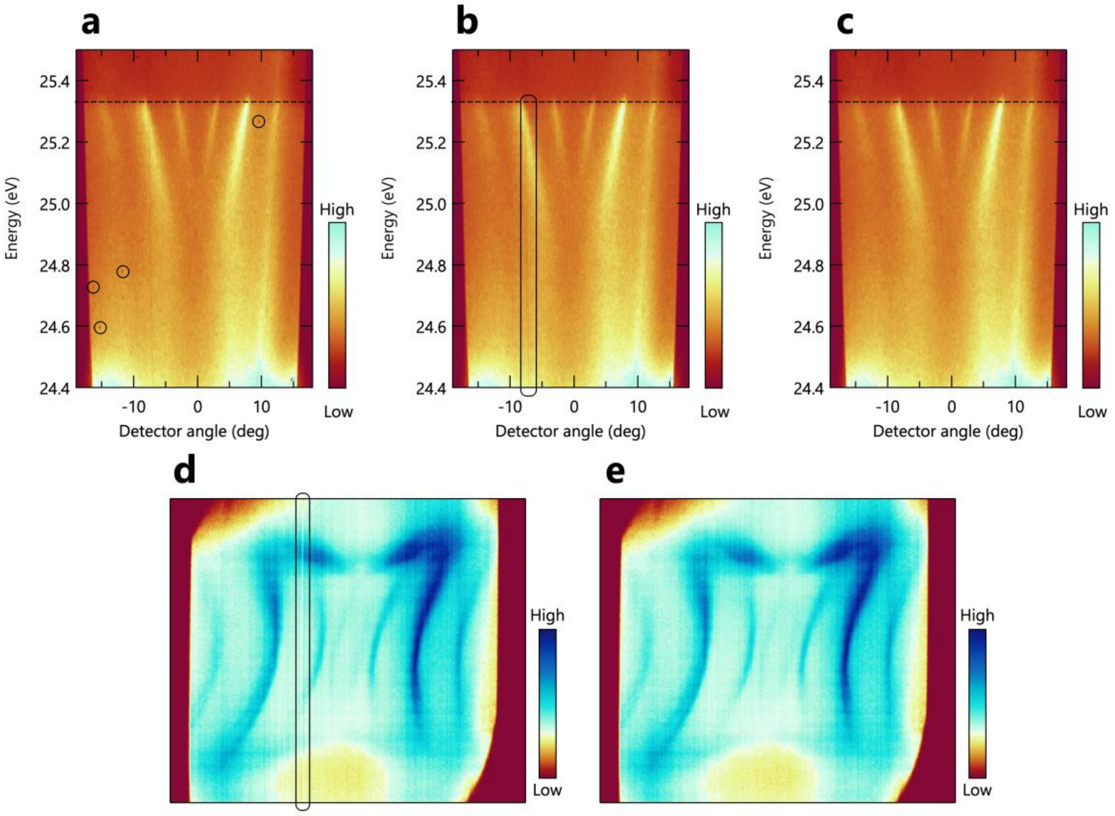

Fig. S1 Image calibration examples on Bi2212 data taken at 30 eV. The raw ARPES images and Fermi surface maps showed extrinsic intensity reductions, which may have been caused by detector damage or other factors. We applied two types of intensity calibrations against block-like and line-like reductions by interpolating the surrounding pixels to enhance visibility. We calibrated both reductions for (**a**) the raw ARPES image and the line reduction for (**d**) the raw Fermi surface map, resulting in (**b**) the block-reduction calibrated ARPES image, (**c**) the both-calibrated ARPES image, and (**e**) the line-reduction calibrated Fermi surface map. The block reductions are represented by circles in (a) for a few exemplary cases, while the line reductions are indicated by rounded rectangles in (b) and (d). Note that we only performed calibrations on the ARPES images and Fermi surfaces, and not on the spin-resolved ARPES data. Therefore, our results and conclusions presented in the main text remain unaffected by these calibrations.
